# Complete Mitochondrial Genomes of the Cherskii’s Sculpin *Cottus czerskii* and Siberian Taimen *Hucho taimen* Reveal GenBank Entry Errors: Incorrect Species Identification and Recombinant Mitochondrial Genome

**DOI:** 10.1177/1176934317726783

**Published:** 2017-08-24

**Authors:** Evgeniy S Balakirev, Pavel A Saveliev, Francisco J Ayala

**Affiliations:** 1 Department of Ecology and Evolutionary Biology, University of California, Irvine, Irvine, CA, USA; 2 A.V. Zhirmunsky Institute of Marine Biology, National Scientific Center of Marine Biology, Far Eastern Branch, Russian Academy of Sciences, Vladivostok, Russia; 3 School of Natural Sciences, Far Eastern Federal University, Vladivostok, Russia

**Keywords:** Cherskii’s sculpin *Cottus czerskii*, Amur sculpin *Cottus szanaga*, erroneous taxonomic identification, Siberian taimen *Hucho taimen*, introgression, recombinant mitochondrial genome, GenBank entry errors monitoring, GenBank Entry Error Depositary

## Abstract

The complete mitochondrial (mt) genome is sequenced in 2 individuals of the Cherskii’s sculpin *Cottus czerskii*. A surprisingly high level of sequence divergence (10.3%) has been detected between the 2 genomes of *C czerskii* studied here and the GenBank mt genome of *C czerskii* (KJ956027). At the same time, a surprisingly low level of divergence (1.4%) has been detected between the GenBank *C czerskii* (KJ956027) and the Amur sculpin *Cottus szanaga* (KX762049, KX762050). We argue that the observed discrepancies are due to incorrect taxonomic identification so that the GenBank accession number KJ956027 represents actually the mt genome of *C szanaga* erroneously identified as *C czerskii*. Our results are of consequence concerning the GenBank database quality, highlighting the potential negative consequences of entry errors, which once they are introduced tend to be propagated among databases and subsequent publications. We illustrate the premise with the data on recombinant mt genome of the Siberian taimen *Hucho taimen* (NCBI Reference Sequence Database NC_016426.1; GenBank accession number HQ897271.1), bearing 2 introgressed fragments (≈0.9 kb [kilobase]) from 2 lenok subspecies, *Brachymystax lenok* and *Brachymystax lenok tsinlingensis*, submitted to GenBank on June 12, 2011. Since the time of submission, the *H taimen* recombinant mt genome leading to incorrect phylogenetic inferences was propagated in multiple subsequent publications despite the fact that nonrecombinant *H taimen* genomes were also available (submitted to GenBank on August 2, 2014; KJ711549, KJ711550). Other examples of recombinant sequences persisting in GenBank are also considered. A GenBank Entry Error Depositary is urgently needed to monitor and avoid a progressive accumulation of wrong biological information.

## Introduction

The Cherskii’s sculpin *Cottus czerskii* Berg 1913^1^ is an amphidromous fish inhabiting the Sea of Japan’s inland coast rivers. The range of the species is limited by the North Nandai River (North Korea) on the South and the Serebryanka River (Primorye Region, Russia) on the North.^1–6^ Recently, Han et al^7^ have published the complete mitochondrial (mt) genome of allegedly *C czerskii* from the Sungari River (the Amur River basin, Heilongjiang Province, China; 47°03′ 39′′N, 128°59′ 33′′E). The previously described range of *C czerskii* did not, however, include the Amur River basin. There were only 2 described sculpin species in the Amur basin, *Cottus szanaga* and *Mesocottus haitej*.^8^ Consequently, we were interested in a comparative genetic analysis of *C czerskii* specimens collected from the Primorye Region, where this species was described originally^1^ and the sample from the Amur River basin investigated by Han et al.^7^

The Siberian taimen *Hucho taimen* Pallas is another fish species, which is considered here in relation to GenBank entry errors. *Hucho taimen* is the world’s largest salmonid fish, reaching up to 2 m in length and 105 kg in weight.^9^ The unique biological features and severe decline of taimen populations have stimulated intensive genetic investigations of the species (Balakirev et al^10^ and references therein). We previously revealed that the GenBank reference sequence of the *H taimen* mt genome (NC_016426.1; accession number HQ897271.1^11^) is recombinant bearing 2 introgressed fragments (around 0.9 kb [kilobase]) from 2 lenok subspecies, *Brachymystax lenok* and *Brachymystax lenok tsinlingensis*.^10^ We sequenced and submitted to GenBank (August 2, 2014; KJ711549, KJ711550) 2 mt genomes of *H taimen* from natural populations of the Amur River basin without introgressions^12^; yet, the recombinant sequence still serves as the GenBank reference sequence of the *H taimen* mt genome.

We describe here GenBank entry errors for 2 fish species, *C czerskii* and *H taimen*. In the case of *C czerskii*, there is reasonable doubt on correct species identification; the data show that the accession number KJ956027 should be listed as *C szanaga* instead of *C czerskii*. In the case of *H taimen*, the mt genome sequence HQ897271.1 appears to be a recombinant sequence including a big chunk of mitochondrial DNA (mtDNA) from 2 lenok subspecies (genus *Brachymystax*) leading to significant biases in phylogenetic inferences.

Our results are of consequence concerning the GenBank database quality, highlighting the potential negative consequences of entry errors, which once they are introduced tend to be propagated among databases and subsequent publications. Taking into account that GenBank entry errors are not rare, a GenBank Entry Error Depositary (EED) is urgently needed to monitor and avoid a progressive accumulation of wrong biological information.

## Materials and Methods

The *C czerskii* specimens were collected from the Barabashevka River (43° 11′51′N, 131° 29′54′E), Primorye Region, Russia. A complete morphological description of *C czerskii* has been performed by one of the authors of this work (P.A.S.).^13^ *Cottus czerskii* differs from all other Palearctic Cottinae by the following complex of features: the presence of teeth on the palatines, a long internal ray of the ventral fin (73.4%-96.0% of the length of the largest ray of the ventral fin), full body seismosensoric canal of 39 to 44 pores passing through the midline of the body, high total number of vertebrae (38-40), and large body size (up to 25 cm in total length).

We have also analyzed the GenBank mt control region (CR) sequences investigated by Yokoyama et al^14^ who collected samples of *C czerskii* from the Barabashevka River. The multiple entries of *C czerskii* (GenBank accession numbers AB308533, AB308534, AB308535, AB308536, AB308537, and AB059350) investigated by Yokoyama et al^14^ are important to visualize the range of intraspecific diversity in the species.

The specimens (Ht5 and Ht16) of the Siberian taimen *H taimen* were collected from the Amur River basin; a specimen of blunt-snouted lenok *Brachymystax tumensis* Mori was collected from the Bikin River (see Balakirev et al.^10^ for sampling locations and procedures). In addition, we used full mt genomes from GenBank (Table S1), which were selected based on previous molecular evidence of close relationship to families Cottidae and Salmonidae and screening of nucleotide sequences available in GenBank.

Total genomic DNA was extracted using the DNeasy Blood & Tissue Kit (Qiagen, Hilden, Germany) from 96% ethanol-preserved muscle tissue. The procedures for DNA amplification and direct sequencing have been described previously.^10,15^ The mt fragments were amplified with primers designed with the program mitoPrimer, v. 1.^16^ The polymerase chain reaction details and primers are presented in Text S1 and Table S2 (online supporting information). The *C czerskii* mt genomes were annotated with the program DOGMA^17^ and deposited in GenBank under accession numbers KY783659 and KY783660.

The mt genomes were assembled using the program SeqMan (Lasergene, DNASTAR, Inc., Madison, Wisconsin, USA). Multiple sequence alignment was conducted using MUSCLE^18^ and MAFFT, v. 7^19^ and manually curated. DnaSP, v. 5^20^ and PROSEQ, v. 2.9^21^ were used for intra- and interspecific comparisons; MEGA, v. 7^22^ was used for basic phylogenetic analyses.^10,15^ For all reconstructions, the best-fit model of nucleotide substitution was chosen with the Akaike information criterion and the Bayesian information criterion in MEGA and jModelTest, v. 2.^23^ The alignments were analyzed for evidence of recombination using various recombination detection methods implemented in the program RDP3.^24^

## Results and Discussion

**Figure 1.**
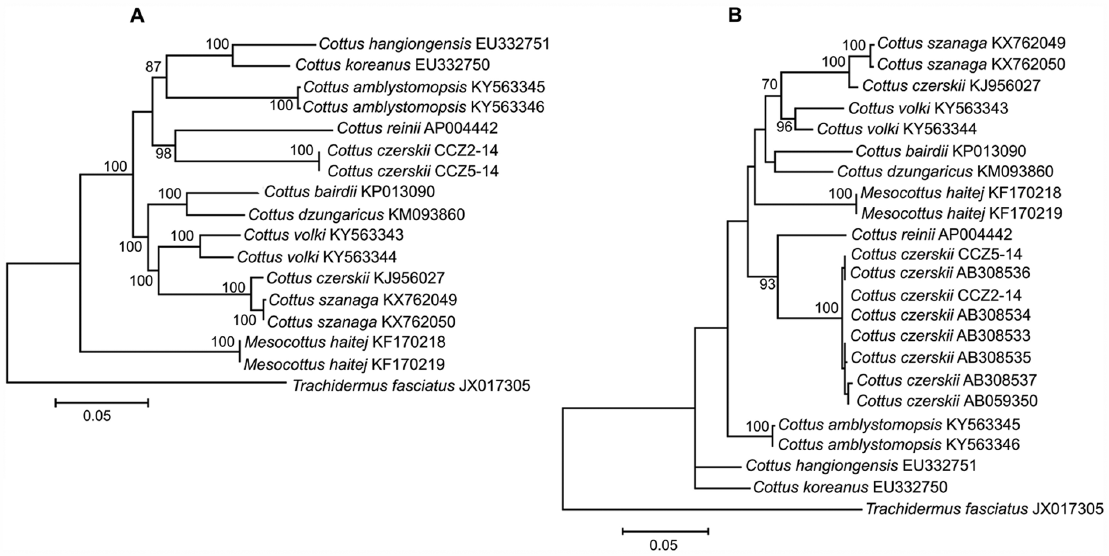
Maximum likelihood trees for the Cherskii’s sculpin *Cottus czerskii* specimens CCZ2-14 and CCZ5-14 and GenBank representatives of the family Cottidae. (A) The trees are constructed using whole mitochondrial genomes or (B) the mitochondrial control region only. The trees are based on the general time reversible + gamma + invariant sites (GTR+G+I) model of nucleotide substitution for whole mitochondrial genomes but Tamura 3-parameter + gamma (T92+G) for the control region separately. The numbers at the nodes are bootstrap percent probability values based on 1000 replications (values below 70% are omitted).

The size of the mt genome of our 2 samples of *C czerskii* is 16 560 bp (base pairs) and the gene arrangement, composition, and size are very similar to the sculpin fish genomes published previously.^25–27^ There were only 6 single nucleotide differences and no length differences between the haplotypes CCZ2-14 and CCZ5-14; total sequence divergence (*D_xy_*) was 0.0004 ± 0.0001. The comparison of the 2 mt genomes now obtained with other complete mt genomes available in GenBank for the genera *Cottus, Mesocottus*, and *Trachidermus* reveals a close affinity of *C czerskii* to other *Cottus* species (Figure 1A). However, a surprisingly high level of sequence divergence (*D_xy_* = 0.1033 ± 0.0030) is detected between the *C czerskii* samples now studied (CCZ2-14 and CCZ5-14) and the *C czerskii* mt genome from GenBank (KJ956027). The average level of mt genome divergence (*D_xy_*) between all 8 *Cottus* available in GenBank (excluding the GenBank *C czerskii*, KJ956027 and our 2 samples), which include *C. bairdii, C. dzungaricus, C. hangiongensis, C. koreanus, C. reinii, C szanaga, C. volki*, and *C. amblystomopsis*, is 0.0907 ± 0.0017. The difference (0.1033) between the mt genomes of *C czerskii* studied here and the previously published GenBank *C czerskii* (KJ956027^7^) is within the range of interspecific level of divergence observed between the 8 listed *Cottus* species. Thus, the mt data indicate that the *C czerskii* sample from the Primorye Region, where this species was described originally, and the sample from the Sungari River and the Amur River basin^7^ are not the same species.

Figure 1A shows the *C czerskii* (KJ956027) from the Sungari River clusters with the Amur sculpin *C szanaga* (KX762049, KX762050) with a surprisingly low level of divergence (*D_xy_* = 0.0135 ± 0.0008), which is in the range of intraspecific mt genome variability in sculpins (about 0.0342 in, eg, *C volki*)^26^. Thus, we may conclude, on one hand, that the GenBank entries for *C czerskii* (KJ956027) and *C szanaga* (KX762049, KX762050), despite their different species names, actually represent the same biological species. On the other hand, 2 entries with the same species name, the previously published GenBank *C czerskii* (KJ956027) and the *C czerskii* now studied, show a surprisingly high level of sequence divergence (0.1033), comparable with the average divergence between other *Cottus* species, clearly indicating that they are different biological species.

The phylogenetic inconsistency we have detected might reflect hybridization event(s) between *C czerskii* and *C szanaga*, which might have resulted in interspecific recombination of their mtDNA (as it has been found for other organisms including fishes^10,15^), or it could be due to incorrect taxonomical identification if it is the case that a specimen of *C szanaga* was erroneously identified as *C czerskii*. We therefore analyzed the mt genome alignments for evidence of recombination using various recombination detection methods implemented in the program RDP3.^24^ All methods failed to reveal any signal of recombination between the GenBank mt genomes of *C czerskii* (KJ956027) and *C szanaga* (KX762049, KX762050) (*P* > .05), thus rejecting hybridization as a possible explanation of the anomalous similarity between the GenBank *C czerskii* (KJ956027) and *C szanaga* (KX762049, KX762050) mt genomes. Thus, the obvious discrepancy in the level of divergence between the mt genome sequences obtained by us and the one downloaded from GenBank is a result of mistaken species identification of KJ956027, so that the specimen investigated by Han et al^7^ actually represents *C szanaga* erroneously identified as *C czerskii*. This conclusion is in accordance with the ichthyologic data describing only 2 sculpin species in the Amur basin, *C szanaga* and *M haitej*^8^; the Cherskii’s sculpin *C czerskii* does not inhabit the Amur River basin.

One more argument supporting incorrect taxonomical identification of KJ95607 as *C czerskii* comes from the analysis of the GenBank nucleotide sequences (mt CR) investigated by Yokoyama et al^14^ who collected *C czerskii* samples from the Primorye Region. Figure 1B shows very close similarity (*D_xy_* = 0.0024 ± 0.0011) between our 2 specimens of *C czerskii* (CCZ2-14 and CCZ5-14) and the specimens investigated by Yokoyama et al.^14^ Moreover, the data sets show 32.9 times higher divergence (*D_xy_* = 0.0789 ± 0.0089) between the GenBank mt genome of the previously misidentified *C czerskii* (KJ956027) and the other *C czerskii* listed in Figure 1B, confirming the analysis based on the complete mt genomes. The intraspecific level of divergence detected between the GenBank CR sequence of the misidentified *C czerskii* (KJ956027) and *C szanaga* (KX762049, KX762050) is *D_xy_* = 0.0170 ± 0.0039. Thus, once again the data show an entry error in the GenBank database so that the accession number KJ956027^7^ should be listed as *C szanaga* instead of *C czerskii*.

Our observations concerning the GenBank database quality highlight a case of potential entry errors, which, once they first appear, tend to be propagated among public databases and subsequent publications (see discussion in the work by Pool and Esnayra^28(p18-20)^). We illustrate this potential error propagation with the salmonid fish Siberian taimen *H taimen* hybrid mt genome below.

**Figure 2.**
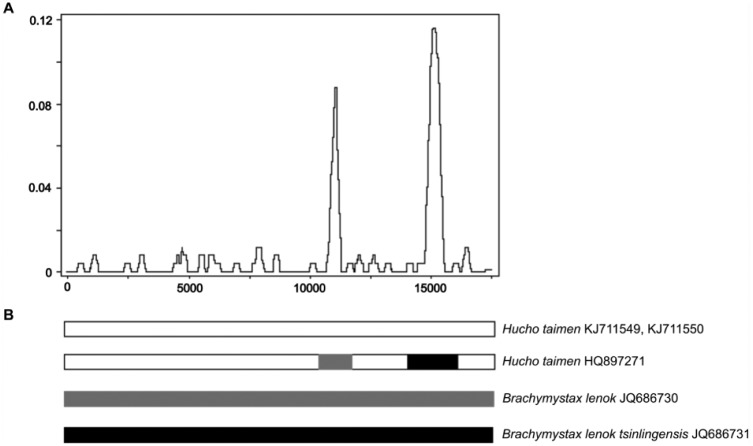
(A) Sliding-window plot of divergence along the complete mitochondrial DNA sequences between the GenBank recombinant *Hucho taimen* mt genome (HQ897271.1) and the nonrecombinant *H taimen* sequences (KJ711549, KJ711550). Window sizes are 250 nucleotides with 25-nucleotide increments. The 2 significant peaks of divergence are centered on 2 genes, *ND3* and *ND6*. (B) Schematic representation of the recombination events in the mitochondrial DNA of GenBank *Hucho taimen* (HQ897271.1). The top parental sequence is from *H taimen* (our study); the 2 bottom parental sequences are from *B lenok* (first recombination event, in gray) and *B lenok tsinlingensis* (second recombination event, in black). Adapted from Balakirev et al.^10^ with modifications.

We have recently sequenced a portion (8141 bp) of the mt genome in 28 specimens of *H taimen* from 6 localities in the Amur River basin.^10^ A comparison of the data with the GenBank *H taimen* mt genome (HQ897271.1^11^) revealed significant differences between them despite the fact that the fish specimens come from neighboring geographical areas. The distribution of divergence was nonuniform, with 2 highly pronounced divergent regions centered on 2 genes, *ND3* and *ND6* (Figure 2A). We have found that the first and second divergent regions are identical between the GenBank *H taimen* and 2 lenok subspecies, *B lenok* and *B lenok tsinlingensis*, respectively. Therefore, both divergent regions represent introgressed mtDNA (~0.9 kb) resulting from intergeneric hybridization between the 2 lenok subspecies and *H taimen*. The 2 recombination events were highly significant (*P* = 2.984 × 10^−25^ and 8.528 × 10^−42^ for the first and second recombination events, respectively^10^) with all 7 methods implemented in the program RDP3.^24^ Introgression is, however, not detected in our *H taimen* specimens (Figure 2B).

Consequently, we sequenced 2 complete mt genomes of *H taimen* from natural populations (the Amur River basin) without introgressions (KJ711549, KJ711550^12^). Yet, the recombinant sequence (HQ897271.1^11^) is used to represent the GenBank mt genome of *H taimen*. It is actively used in phylogenetic inferences,^29–37^ which in turn have been cited in at least 48 subsequent publications (Google Search, July 7, 2017). It is worth noting that the phylogenetic inferences based on recombinant genes and genomes are significantly biased.^10,15^

**Figure 3.**
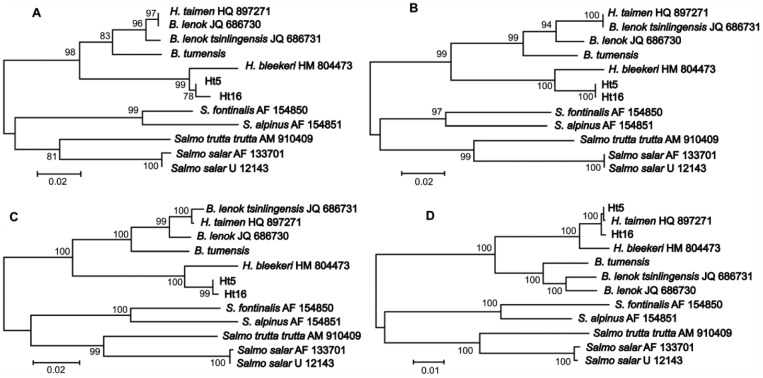
Phylogenetic trees of *Hucho taimen* and its relatives based on different fragments of the mitochondrial DNA sequences: (A) first introgressed region only (*ND3* gene, see text and Figure 2A), (B) second introgressed region only (*ND6* gene), (C) first plus second introgressed regions (*ND3* + *ND6* genes), and (D) without the 2 introgressed regions. The tree topologies obtained with maximum likelihood and Bayesian inference are congruent (see Balakirev et al^10^ for details). Two sequences of *H taimen*, Ht5 and Ht16 (GenBank accession numbers KJ711549, KJ711550), representing haplotype groups 1 and 2, are included. The *Salvelinus* and *Salmo* sequences (see Table S1 for details) are used as outgroups. Note the changed position of GenBank *H taimen* (HQ897271), depending on the region used for the tree reconstruction. Adapted from Balakirev et al.^10^

Figure 3 illustrates sharply discordant phylogenetic signals between recombinant genome of *H taimen* (HQ897271) and *Brachymystax* subspecies. As a consequence, the position of *H taimen* (HQ897271) was sharply different, depending on the fragments used for tree reconstruction. The trees based on first and second introgressed fragments separately showed *H taimen* (HQ897271) identical to *B lenok* (JQ686730) or to *B lenok tsinlingensis* (JQ686731), respectively (Figure 3A and B). The tree based on both introgressed fragments displayed *H taimen* (HQ897271) between the 2 lenok species (Figure 3C). On the tree excluding the introgressed fragments, *H taimen* (HQ897271) was within the same cluster as the other *H taimen* specimens (Ht5 and Ht16; Figure 3D). Thus, most of the mt genome of *H taimen* (HQ897271) has obvious similarity to the *H taimen* sequences obtained in our study (the specimens Ht5 and Ht16), whereas the introgressed fragments have unexpected similarity to *Brachymystax* subspecies and could be explained by introgression of mtDNA resulting from hybridization between lenok and taimen. Other salmonids included in this analysis (*Salmo salmo, Salmo trutta, Salvelinus fontinalis*, and *Salvelinus alpinus*) did not show any visible discordance in the level of divergence between the introgressed fragments and the rest of the mt genome (Figure 3).

**Figure 4.**
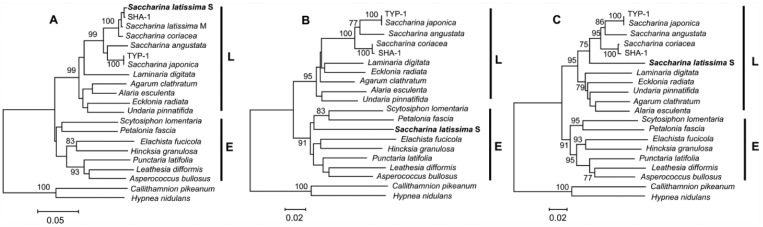
Phylogenetic trees of the brown alga *Saccharina latissima* and its relatives based on different fragments of the mitochondrial *COI* gene (GenBank accession number EU681420): (A) 5’-*COI*, (B) 3’-*COI*, and (C) full *COI* region. 5’-*COI*: the 658-bp (base pair) fragment covering the 5’-flanking region of the *COI* gene. The region starts 123 bp downstream of the *COI* start codon and ends 822 bp upstream of the *COI* stop codon. This fragment has been recommended for algae “barcoding” (species identification).^43^ 3’-*COI*: the 597-bp fragment covering the 3’-flanking region of the *COI* gene that starts 781 bp downstream of the *COI* start codon and ends 225 bp upstream of the *COI* stop codon. Full *COI*: the 1378-bp fragment covering most of the *COI* gene. The fragment starts 123 bp downstream of the *COI* start codon and ends 225 bp upstream of the *COI* stop codon (the full *COI* region represents the largest sequence available for laminarialean algae in GenBank, excluding species for which full mtDNA sequences have been obtained). Representative sequences of the orders Laminariales (L) and Ectocarpales (E) included in these trees are marked by vertical lines. Red algae *COI* sequences of *Callithamnion pikeanum* and *Hypnea nidulans* (GenBank accession numbers EU194965 and FJ694907, respectively) are used as outgroups. Note the changed position of *S latissima* (in bold) depending on the *COI* region used for the tree. The *S latissima COI* sequence denoted as “S” is from Silberfeld et al.^42^ See Additional Table S1 for GenBank accession numbers. Adapted from Balakirev et al.^15^

Instances of interspecific mtDNA recombination have been occasionally detected in hybridizing conifers,^38^ salmonids, *Salmo* and *Salvelinus*,^39,40^ and primates.^41^ We, however, conjecture that the number of recombinant sequences persisting in GenBank could be higher if they are mostly indistinct with basic phylogenetic analysis. For instance, we previously detected unrecognized (“cryptic”) recombinant *COI* genes in 2 brown algae, *Saccharina latissima* (EU681420) and *Cystophora retorta* (GQ368259).^15^ These cryptic recombinants were not detected in the original publication.^42^ However, we showed^15^ that the recombinant sequences have drastic consequences in phylogenetic inferences. Figure 4 shows an example for *S latissima* phylogenetic reconstructions based on recombinant *COI* sequences. The position of *S latissima* on 5′-*COI* and 3′-*COI*–based trees are sharply different; on the 5′-*COI*–based tree, *S latissima* is within the order Laminariales (Figure 4A). On the 3′-*COI* tree, *S latissima* is significantly different from Laminariales algae and clusters with some species of the order Ectocarpales (Figure 4B). On the full-length *COI* tree, *S latissima* is within the order Laminariales (Figure 4C) but significantly different from other *Saccharina* species. Using various recombination detection methods implemented in the program RDP3,^24^ we showed that the *COI* sequence in *S latissima* is recombinant with the parental *COI* sequences of *S latissima* come from different algae orders, Ectocarpales and Laminariales.^15^ The recombinant *COI* sequences are from a highly cited paper^42^; 138 citations; Google Search, July 7, 2017), which potentially might introduce significant biases in subsequent phylogenetic analyses. Our results are relevant concerning the DNA “barcoding” for algae and possibly other organisms. The 5′-*COI* “barcode” region is not representative and might be even misleading (at least in case of *S latissima* and *C retorta*) in resolving taxonomic relationships between algal species.

Thus, entry errors are indeed progressively multiplied, thus propagating incorrect biological information. We may note that entry errors are not easy to remove from the GenBank database.^28^ Even if an error is corrected, it may have been already multiplied in computational analyses by GenBank users, who have already downloaded the erroneous data. One possible approach to reduce the flow of incorrect information is to establish a GenBank EED, where all known entry errors should be collected. The EED should then be first visited by GenBank users so as to identify possible entry errors that may have been reported earlier concerning the genes and/or species of interest, which would avoid their continuing propagation.
